# Sulfur Geochemistry of a Lacustrine Record from Taiwan Reveals Enhanced Marine Aerosol Input during the Early Holocene

**DOI:** 10.1038/srep38989

**Published:** 2016-12-12

**Authors:** Xiaodong Ding, Dawei Li, Liwei Zheng, Hongyan Bao, Huei-Fen Chen, Shuh-Ji Kao

**Affiliations:** 1 State Key Laboratory of Marine Environmental Science, Xiamen University, Xiamen, 361102, China; 2 Institute of Applied Geosciences, National Taiwan Ocean University, Keelung 20224, Taiwan

## Abstract

Lacustrine record of marine aerosol input has rarely been documented. Here, we present the sulfur geochemistry during the last deglaciation and early Holocene of a sediment core retrieved from the Dongyuan Lake in southern Taiwan. An unusually high sulfur peak accompanying pyrite presence is observed at 10.5 ka BP. Such high sulfur content in lacustrine record is unusual. The δ^34^S of sulfur varied from +9.5 to + 17.1‰ with two significant positive shifts at 10.5 and 9.4 ka BP. The sources of sulfur and potential processes involving the sulfur isotope variation including bacterial sulfate reduction, volcanic emissions, in-catchment sulfide oxidation and marine aerosol input are discussed. Enhanced marine aerosol input is the most likely explanation for such sulfur peaks and δ^34^S shifts. The positive δ^34^S shifts appeared concurrently with the maximum landslide events over Taiwan resulted from enhanced typhoon activities. The synchronicity among records suggests that increased typhoon activities promoted sea spray, and consequently enhanced the marine aerosol input with ^34^S-enriched sulfate. Our sulfur geochemistry data revealed sea spray history and marine influence onto terrestrial environment at coastal regions. Wider coverage of spatial-temporal lacustrine sulfur geochemistry record is needed to validate the applicability of sulfur proxy in paleoenvironmental research.

Lacustrine sediments archived environmental history, from which hydrological cycles, erosion and vegetation evolution have been successfully inferred from various proxies^1^. However, only limited documentations were reported regarding the history of sea spray or marine aerosol input^2^. Sulfate, a major ion in seawater and marine aerosol but normally present only in trace amounts in freshwater lakes, makes sulfur a potential proxy to study past environment changes^3^,^4^,^5^,^6^,^7^,^8^,^9^,^10^,^11^. However, the complexity of the sulfur cycle and the scarcity of studies of the behaviour of sulfur in heterogeneous lakes hampered the applicability of using sedimentary sulfur geochemistry to construct past environmental conditions^7^. Stable sulfur isotope is a potential tracer for sulfur sources, but research on the sulfur isotope in lake sediments has focused on the human perturbations on sulfur cycle and ecosystem acidification due to amount of anthropogenic SO_2_ emissions over the last the 200 years since the beginning of the Industrial Revolution^12^,^13^,^14^,^15^. Only a few studies have been conducted for long term environmental changes, e.g., on the glacial-interglacial time scale, by using sulfur isotopic compositions in lacustrine sediments^2^,^6^,^16^.

The sulfur isotopic ratios of sulfate among the different sources that access aquatic environments are variable yet separable. It has been tested that the δ^34^S of sulfate varied in a narrow range (+21‰ ± 0.2‰)^17^ regardless of the water depth, temperature and salinity. The oceanic sulfate via sea spray also have δ^34^S values close to the sea water sulfate^18^. The majority of δ^34^S values in freshwater sources range between 0 and +10‰^19^. The sulfate ions in freshwater that originate from terrestrial sources would reflect the eroded and soluble parts of the rocks, soils and minerals from the local region^20^. The sulfur isotopic compositions in the rain and snow reflect a mixture of volatilized water from different areas covering diverse geological backgrounds and other environmental sources (e.g., soil and rock erosion, aerosols, and dust)^18^. Volcanic eruption also contributes to the atmospheric deposition of sulfur^21^,^22^,^23^,^24^,^25^. The δ^34^S values of volcanic gaseous sulfur range from −15‰ to +13‰ and cluster around 0‰^26^. The distinctive isotopic signatures of sulfate sources allow us to discern potential sources of sulfur supplied to the lakes.

In this study, we investigated the sulfur geochemistry including isotopic composition of a sediment core, covering the last deglaciation and the Early Holocene, retrieved from the Dongyuan Lake in southern Taiwan (Fig. 1). In Dongyuan Lake, we observed significant variation in the sulfur content, with a peak value up to 4.8%, accompanied by pyrite occurrence, which is unusual in freshwater lakes^27^. By using the isotopic compositions of sedimentary sulfur, we unravelled the marine influence of sulfur source on a freshwater lake and linked the variation in sulfur isotopes to climate changes in the western Pacific.

## Results

The down-core variation in the total sulfur (TS), C/S ratio, δ^34^S of sulfur and major elements (Fe, Al, Na and K) are illustrated in Fig. 2. Within the investigated section, the content of TS varied from 0 to 4.82% (Fig. 2), revealing prominent variations. The variation pattern generally followed the temporal pattern of the total carbon (TC; from 0.35 to 19.25%) reported previously^28^. An extraordinary peak of TS (>1%), which is very unusual in a freshwater lake, was observed at ~10.5 ka BP in accordance with the TC peak. In addition, X-ray diffraction (XRD) results showed the presence of pyrite (FeS_2_) minerals in the specific layer (Fig. 3). Abundant wood debris was also observed in this specific time period^28^. The TC variation was mainly attributed to the fluctuation of the organic matter input from the catchment reflecting the East Asian summer monsoon rainfall variation^28^,^29^.

The C/S ratio fluctuated between 2 and 65, remarkably, with large amplitude during the early Holocene (Fig. 2). As the organic C/S ratio is near 50, higher ratios may suggest the control of organic S and lower values may result from the addition of inorganic S. The lowest C/S ratio (~2.5) appeared correspondingly with the TS peak at ~10.5 ka BP suggesting an extra addition of sulfur.

The variation of the δ^34^S values in the Dongyuan Lake sediments ranged from +9.5 to +17.1‰ (Fig. 2). The δ^34^S values decreased from+ 13 to +9.5‰ from 17 to 14 ka BP and then rapidly increased to +17.1‰ at 9.4 ka BP. Two significant peaks in δ^34^S can be observed at 10.5 ka BP and 9.4 ka BP (Fig. 2). All these positive shifts in δ^34^S were concurrent with the peaks of TC and TS.

The total iron concentration varied in a narrower range from 1.6 to 3.5% (excluding the iron peak) with an average value of 2.4% ± 0.5%. The aluminium concentration varied irregularly between 5.9 and 11.1% with an average value of 8.7% ± 1.0%. The sodium concentration varied between 0.19 and 0.46% with an average value of 0.37% ± 0.04%. The potassium concentration varied between 1.25 and 2.63% with an average value of 2.08% ± 0.24% (Fig. 2). There was no significant correlation between δ^34^S and Al concentrations suggesting that the variation in δ^34^S was not linked to the input of lithogenic material. Meanwhile, the temporal variation of iron did not follow the variation of the aluminium, suggesting that the iron was more chemically dynamic and its variation was not controlled by the lithogenic input.

## Discussion

### C-S-Fe relationships and the sedimentary environment

The relations among sedimentary C, S and Fe can be utilized to examine the post-depositional geochemical condition^7^,^30^,^31^. Almost all of the sulfur in nature lakes is delivered as sulfate originating from weathering and oxidation reactions in the lake catchment^7^. Sulfate in the water column that diffuses into the sediments will be utilized by sulfate reducing bacteria to oxidize organic matter under anoxic conditions^32^. Sedimentary sulfate reduction produces H_2_S, which accumulates in pore water and may diffuse back to the water column to be re-oxidized. However, in the presence of ferrous iron, which is produced by the reduction of iron oxide, H_2_S precipitates to form iron sulfide (e.g., pyrite) and is eventually buried in the sediments^33^. Thus, when the ferrous iron is sufficiently high the sedimentary system is a closed system for H_2_S.

Compared to marine environments, sulfate concentration in fresh water lakes are generally low while the rate of iron supply to the lake sediments are typically high^7^. The scatter plot of TS vs. the total iron concentration (Fig. 4a) shows that all of the measured data points fall on the right-hand side, with much lower TS/Fe ratios compared with that of pyrite. Such low TS/Fe ratios suggest that the sedimentary iron in our system was sufficiently high to precipitate the H_2_S generated by bacterial sulfate reduction. The iron in our system was still sufficient even if 50% of the reactive iron in the total iron is assumed to be available for sulfide formation.

The ratio of sedimentary S to organic C has been widely used to distinguish marine and freshwater sediments^27^,^34^,^35^,^36^. The C–S slope of sediment samples in Dongyuan Lake lies along the freshwater trend^27^ (~0.05, Fig. 4b), except for those observed during the extraordinary peak at ~10.5 ka BP, when the pyrite was detected (Fig. 3). These sediment samples with TS >1% at around 10.5 ka BP deviate significantly from the C-S trend for the fresh water system and approach the ratio for the marine environment^27^ (C–S slope = ~0.36, Fig. 4b).

Meanwhile, the intercept of the C-S plot may reveal information of water column and sedimentary sulfate reduction. If sulfate reduction occurs in the water column, the intercepts of the C-S plots should be positive^6^,^37^. In general, the vertical mixing of the water column is relatively strong in small and shallow lakes in Taiwan due to the frequent tropical cyclone in summer and strong northeast monsoon winds in winter. Thus, the water column sulfate reduction is limited. This is also supported by the C-S plot with nearly zero intercepts (Fig. 4b). Accordingly, the sulfate reduction happens mainly in the sediments after deposition.

Overall, the C-S-Fe relationships suggest that the bacterial sulfate reduction should happen in the sediments instead of in the water column, and the sediment in Dongyuan Lake is a closed system that efficiently traps the H_2_S generated by the bacterial sulfate reduction.

### Evaluation of sources and processes of sulfur supplied to the lake by δ^34^S

To trace the source of the sulfate using sulfur isotopes, the biological fractionation needs to be evaluated first. When the concentrations of aqueous sulfate is high, the sulfur isotope fractionation during reduction is quite large ranging from 16 to 42‰^38^. The isotope fractionation by the bacterial sulfate reduction is 28‰ on average^38^ and is most pronounced when the sulfate concentration is greater than 2 mM^39^. However, the sulfate concentrations in freshwater lakes are typically low^27^. Moreover, Dongyuan Lake was generally depleted in S compared to iron (see in 4.1, Fig. 4a), suggesting a closed system. Thus, the δ^34^S of the sulfide resulting from the bacteria sulfate reduction would be completely trapped, and the δ^34^S values would be close to those of the original sulfate. The fractionation of the bacteria sulfate reduction in Dongyuan Lake was likely a minor factor for the observed changes in δ^34^S in our record.

The sources of sulfur in Dongyuan Lake are limited because of the absence of sulfur rich bedrock sources (the bedrocks are mainly composed of shale, siltstone and argillite). However, the oxidation of the iron sulfide minerals inherited in bedrocks might not be low. A sulfide oxidation rate of 7.8 ± 0.6 × 10^9^ mol of SO_4_^2−^ y^−1^ is estimated for the Kaoping basin in Southwestern Taiwan (~400 times the global mean sulfide oxidation per area)^40^. The warm and wet climate is responsible for such a high sulfide oxidation. The δ^34^S of sulfate in the river water of Kaoping ranged from −9.0 to +3.2‰, and ~90% of the collected water samples were characterized by negative values^40^. Since the bedrock of Dongyuan Lake shares a similar lithology to the Kaoping watershed^41^, we anticipate negative δ^34^S values for the sulfate that drained into our study lake. Moreover, it has been reported that seventeen out of nineteen samples from a 200-meter long sediment core drilled in the coastal plain of Southwestern Taiwan feature negative δ^34^S values, with the sample closest to the surface having a δ^34^S of −8.3‰ and the samples representing the onset of the Pleistocene epoch having δ^34^S values around −6‰^42^. All above mentioned evidence suggests that the sulfate leached from bedrock via oxidation of sulfide minerals cannot explain the significant positive δ^34^S values in our records. The much positive δ^34^S values in our record also suggest that the marine influence on the sulfur geochemistry of freshwater lakes on subtropical islands cannot be overlooked.

In addition to the weathering of sulfide minerals, the sulfur might also come from atmospheric deposition or rainfall. As mentioned above, volcanic emission is a potential source of atmospheric sulfate. The contribution of volcanic sources to the atmospheric sulfur cycle is important on the global scale. The annual and global mean contribution to the wet deposition flux from volcanic sources was suggested to be up to 30%^25^. The archived volcanic activities inferred from sulfate flux preserved in the Greenland ice core 2 (GISP2)^43^ (Fig. 5a) do not show any sulfur emission peaks corresponding to the sulfur and sulfur isotope peaks in our lake. Additionally, there is no active local volcano in the past 20 ka that could have supplied volcanic sulfur to the study area. Although the long-distance transport of SO_2_ or tephra from volcanoes in Japan or Korea may not be ignored, the chemical composition of the tephra from the only eruption nearby in time (Ulleung-Oki, 10.7 ka BP)^44^ was enriched in K and Na, which cannot be supported by our narrow ranged and less variable Na/Al and K/Al ratios (Fig. 5b). The Na/Al ratio in the volcanic glass of U-oki (0.53) is ~10 times higher than the ratios observed in the Dongyuan Lake sediments (<0.06; Fig. 5b). Thus, the influence from the U-Oki eruption was also unlikely. Furthermore, our sulfur and sulfur isotope peaks appeared concurrently with amounts of terrestrial organic input and wood debris that were previously attributed to strong typhoon activities^28^. The terrestrial organics sulfur peaks were unlikely a coincidence, thus, we exclude the global and regional volcanic activity as an important control on δ^34^S variations although the volcanic activity may make substantial contribution to the global sulfur budget.

**Figure 1:**
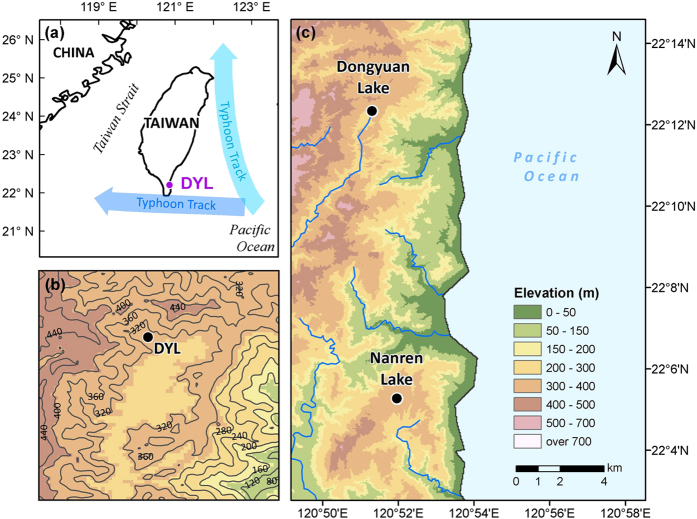
Study area. (**a**) Location of Dongyuan Lake (DYL) in southern Taiwan. The historical typhoon tracks are indicated. (**b**) Topography of Dongyuan Lake around Dongyuan Lake (DYL). (**c**) Geographical and topographical features of Dongyuan Lake and a nearby Naren Lake (see Methods). This map (including **a**,**b** and **c**) was created using ArcGIS 10.3.1 software (ESRI Corporation, Redlands, California, USA, https://www.arcgis.com/).

**Figure 2:**
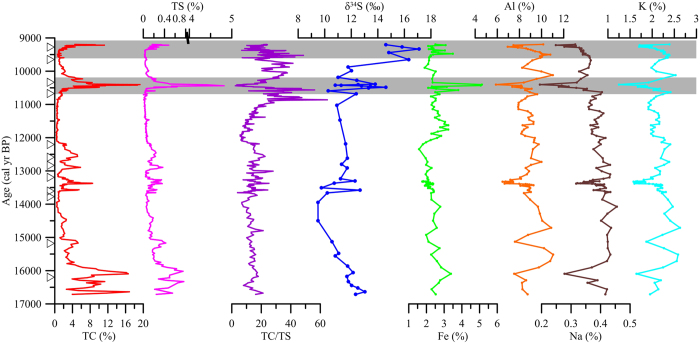
Down core profiles of TC, TS, TC/TS, δ^34^S, Fe, Al, Na and K. The shaded grey bars highlight the major shifts in the sulfur isotope. The triangles indicate the ^14^C dates used for the age model. The data of TC was reported by Ding *et al*.^28^.

**Figure 3:**
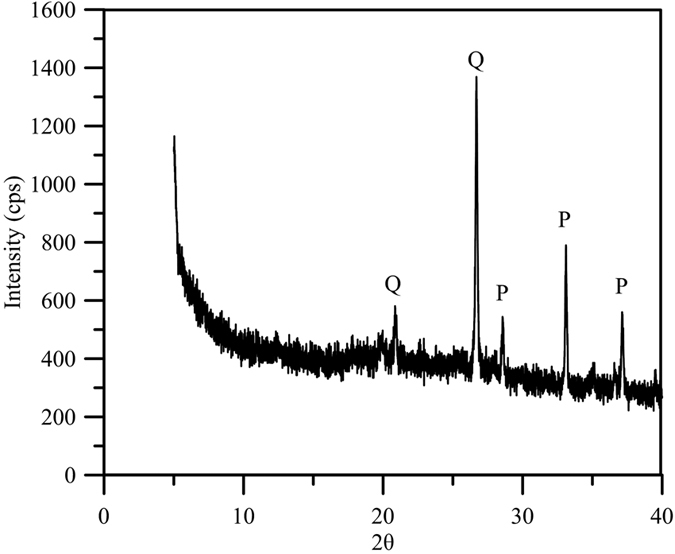
X-ray diffractograms of the sediment sample around 10.5 ka BP. Q, quartz; P, Pyrite.

**Figure 4:**
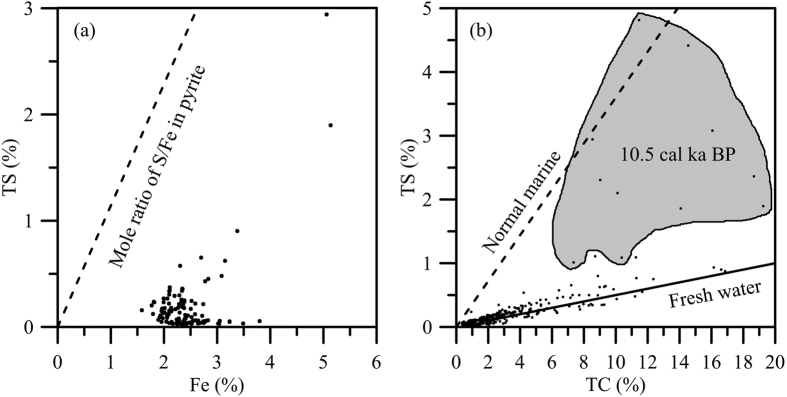
Scatter plots of TS vs. Fe (**a**) and TS vs. TC (**b**) in Dongyuan Lake sediments. The dashed line in (**a**) marks the molar ratio of pyrite. The shaded area in (**b**) indicates the samples located at ~10.5 cal ka BP. Dashed and solid lines in (**b**) are regression lines for normal marine (oxygenated bottom water) and freshwater lakes, respectively^27^.

**Figure 5:**
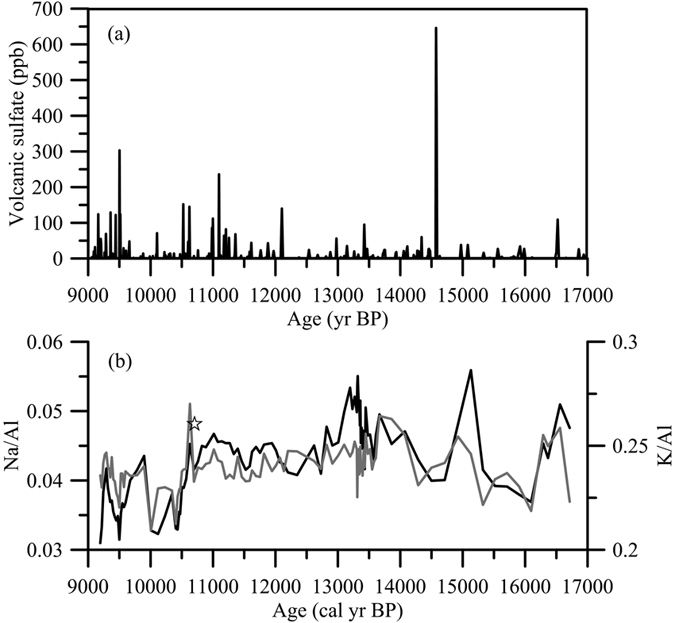
(**a**) Time serie of volcanic sulfate records in the GISP2 core^43^. (**b**) Na/Al (black line) and K/Al (grey line) in the sediments of Dongyuan Lake. The star represents the time of the volcanic eruption of U-oki.

In fact, the high sulfur content in inland freshwater lakes adjacent to coastal zones has been attributed to sea salt spray when bedrock sources were not significant^8^,^9^,^10^,^11^,^27^. The sulfur content can reach 8% due to the influence of sea salt spray^10^. The sea spray sulfates in aerosols and rain have δ^34^S values of +21‰^45^. The influence of the seawater-derived sulfate decreases rapidly as it travels inland^46^. For a 2-year monitoring period in the British Isles, the δ^34^S value of atmospheric deposition averaged +16, 14, and 3‰ at 0, 1, and 55 km landward from the shoreline^47^,^48^. The influence of the sea salt sulfates on the δ^34^S values of soils against distance to the coast has also been tested. The effect from marine aerosols reach up to 30 km inland, as evidenced by high coastal soil δ^34^S values (+18‰) and less positive values (+10‰) for inland soil^49^,^50^. At greater distances, there was no detectable contribution from marine sulfate to the soil δ^34^S values^18^. Similar effects were observed for river sulfate δ^34^S values which gradually increased from 2.25‰ at upstream to 16.3‰ at the coast due to the sea spray influence within 30 km^51^. Given Dongyuan Lake’s proximity to the sea (~4 km, Fig. 1) and the fact that the bedrock contains limited sources of sulfur, the high δ^34^S values (from +9.5 to +17.1‰) in Dongyuan Lake likely reflect the relative input of the sea-spray aerosol (+21‰) and continental pre-anthropogenic atmosphere (+8‰)^2^. Moreover, the modern water chemistry in a near-by freshwater lake also suggests that the investigated lake is significantly influenced by sea salt spray (see Methods).

**Figure 6:**
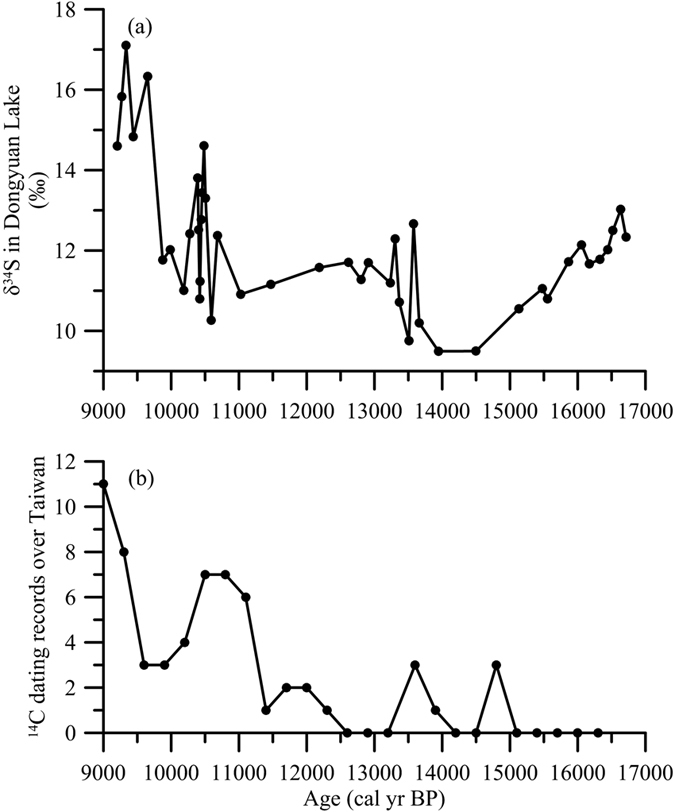
Comparison between δ^34^S record in Dongyuan Lake (**a**) and ancient landslide records over Taiwan^54^ (**b**). The ^14^C dating record is expressed as the total number of ^14^C dates of landslides within a 300-yrs interval.

Taiwan’s climate is significantly influenced by typhoons. Earlier studies have reported that large quantities of sea salt particles, including sulfate, can be transported to the land by episodic typhoon events^52^,^53^. Very likely, the ^34^S-enriched sulfate was carried by tropical cyclones. We recently reported an intimate relation between the peak organics and the occurrences of ancient landslides archived in the floodplain over Taiwn^28^. Based on the distinct correlation, we speculate that the relatively high sulfur content and the positive shifts in δ^34^S at 10.5 ka BP and 9.4 ka BP were caused by enhanced marine aerosol input. We compared our δ^34^S and the island wide landslide dating record^54^ (landslide frequency) in Fig. 6. It is interesting to see that the most pronounced δ^34^S peaks were correlated well with the frequency of landslide occurrence on the river floodplains (Fig. 6). The maximum landslide events occurred at 10.5 ka BP and 9.4 ka BP. The two events, triggered by significantly increased typhoon activities, are much larger than any other events in the past 15 ka^54^. Thus, we infer that the input of marine sourced sulfate at around 10.5 ka BP and 9.4 ka BP increased greatly due to the increased typhoon activities. The pulses of the sulfur content and sulfur isotopes that occurred during the early Holocene are likely associated with the highest solar insolation, maximum sea surface temperature (SST) plateau in the western tropical Pacific^23^ and maximum East Asian summer monsoon during the early Holocene^54^. It has been reported that both the frequency and intensity of typhoons increase when the tropical Ocean SST increases^55^. We suspected that the highest solar insolation and western tropical Pacific SST during the Early Holocene might have led to more frequent and more intense typhoons, which in turn, brought sea salt onto the land.

So far, no proper index for sea spray has been established. We are aware that wind intensity is the primary forcing, but it is not necessarily caused by episodic typhoons. Relatively higher TS and δ^34^S values at around 16.5 ka BP are also observed in our record. These values might not be driven by the aforementioned typhoon activities (Fig. 6). During this specific period, Heinrich Stadial 1, prevailed cold Northern hemisphere climate may have stronger winter monsoon wind, which might have stirred up droplets of seawater into the air and brought marine aerosol into the lake. The lowest δ^34^S values around 14.5 ka BP (+8‰) might suggest less influence of marine aerosols during that time period.

### δ^34^S comparison between Dongyuan Lake and other lakes

It has been reported that the increased sulfur contents were in accordance with significant positive shifts of δ^34^S (from +3.7 to 32.6‰) in Lake Hovsgol (northwest Mongolia) during the last glacial-post glacial transition^6^. The amplitude of the variation in δ^34^S of their record was much higher than ours. They suggested that the lake received remarkable amount of ^34^S-enriched sulfate during that period, and the ^34^S-enriched sulfate may have accumulated on the lake shore through bacteria sulfate reduction during the last glacial. Increases in the precipitation and glacial meltwater influx and lake level increases during the last deglaciation could have contributed to the large supply of ^34^S-enriched dissolved sulfate into Lake Hovsgol. However, the lake was an inland continental setting in Mongolia and would not have been influenced by marine aerosols.

Recently, the S isotope stratigraphy during the late Pleistocene and Holocene of Lake Tulane, Florida, USA, was investigated^2^. The variation in δ^34^S in the Lake Tulane sediment ranged from +7.3 to +14.7‰, similar to the variation range of our record. In the Lake Tulane sediment, the variability of δ^34^S was suggested to result primarily from the interplay between continental S source (isotopically lighter) and oceanic S source, with the latter one was influenced by the sea level modulated distance to the source of marine aerosol^2^. In Taiwan, the distance to the marine source remained unchanged throughout the last glacial cycle due to Taiwan’s unique setting (no shelf on the eastern shore of Taiwan). Thus, the enrichment of TS and ^34^S in our record may simply reflect an enhancement of the marine aerosol input.

We provided the first long record in Southeast Asia about the marine influence on terrestrial freshwater system as well as the sulfur isotope linkage to ocean-climate dynamics in the western North Pacific, which may advance our knowledge about lacustrine sulfur biogeochemistry at the land ocean boundary. We anticipate that more lacustrine sulfur records will be reported, with additional geographical coverage, we may have a better chance to pin down the spatial scale of the sea spray influence. More studies of sulfur geochemistry in freshwater lake are needed to validate the applicability of the sulfur proxy in paleoenvironmental research.

## Methods

### Study site

Dongyuan Lake is a freshwater lake (22°10′N, 120°50′E) located at 360 m altitude on the east coast of the Hengchun peninsula, southern Taiwan (Fig. 1). The lake has a surface area of 2 × 10^4^ m^2^ and the catchment area covers approximately 94 × 10^4^ m^2^ from elevation of 360 m to 500 m above sea level. The humid tropical and subtropical climate in Taiwan is very much influenced by the East Asian summer monsoon and tropical cyclones in summer season. The contemporary average monthly air temperatures in the study area vary between 20.7 and 28.4 °C with an annual mean temperature of 25.1 °C. The monthly precipitation ranged from 20 mm in January to 460 mm in August with annual precipitation more than 2000 mm, approximately 90% of which occurs during the summer (May to October). During the winter months, relatively cool and dry climate dominated and northeasterly winter monsoon wind prevails from October till April the next year. Since the lake faces the western Pacific, unlike many inland lakes, it may receive sea salt. Most importantly, Taiwan lies along the so called “Typhoon Alley”. More than half of the rainfall of Taiwan comes from episodic typhoons coming from the Western Pacific. On average, three to four typhoons pass over Taiwan every year (Fig. 1). Typhoons bring both rainfall and strong winds and waves toward the east coast of Taiwan.

Since the Dongyuan Lake had been disturbed by human activities and the water chemistry data are not representative, we referred to a near-by lake, Nanren, in a national park (Fig. 1c). Lake Nanren is relatively pristine and shares similar environmental conditions at the same altitude (320 m asl.). The pH value of the lake water ranges from 5 to 7, which is common in Taiwan due to the generally low pH values of the soils (4.5–5.1). The most abundant major ions are reported to be Na^+^and Cl^−^ (0.619 meq/l and 0.677 meq/l respectively), suggesting that the lake is significantly influenced by sea spray^56^.

### Core acquisition and chronology

The sediment core of TYP-B was collected from the centre of Dongyuan Lake, southern Taiwan in 2004. The lithological composition of the core consists of alternating layers of grey-green or dark brown muds and dark grey muds containing amounts of vascular plants which have been described previously^28^. The chronology of the entire core was established previously with 16 AMS ^14^C on organic materials of plant debris^29^. Later, we added 12 more radiocarbon dates specifically for the period of the last deglaciation and the early Holocene to obtain a better chronology^28^. The conventional ^14^C ages were then converted to calendar years (calibrated ages) using the INTCAL13 data set^57^.

### Chemical analyses

For the total sulfur and carbon analyses, 460 bulk ground samples with 1-cm intervals were determined using a HORIBA EMIA model CS500 analyzer with a resistance furnace and a ND-IR detector. Approximately 0.2 g of sample was combusted in the analyser to produce SO_2_ at 1350 °C. Such a high temperature with sufficient oxygen supply ensures a complete combustion of pyrite. The relative precisions for TS and TC analyses are better than 1%. The TC data have been reported previously^28^, in which TOC and TC were reported to be highly correlated due to negligible amount of carbonate in the entire core. The inorganic carbon was less than 0.2% based on calculations with previously published Ca data (assuming that all Ca was in the form of CaCO_3_).

The sulfur isotope ratio (δ^34^S) was determined by using continuous flow elemental analyser connected to an isotope ratio mass spectrometer (Isoprime Ltd, UK). The results are expressed in δ^34^S notation, as a ‰ deviation of the ^34^S/^32^S ratio in the sample from the V-CDT standard. The measurements were directly calibrated using an International Atomic Energy Agency standard (IAEA-S-4, 16.9‰). The standard deviation of the δ^34^S measurements was less than 0.2‰.

Selected samples (5-cm interval) were analysed for sodium, aluminium, potassium and iron contents. Approximately 0.1 g pulverized sediment sample were digested using an acid mixture of Supra-pure HF, HNO_3_ and HClO_4_ (see detail in ref. 58). The concentrations of sodium, aluminium, potassium and iron in the digested solutions were measured using an ICP-OES (Optima 3200DV, Perkin-Elmer^TM^ Instruments, Waltham, Massachusetts, United States). The accuracy and precision were within 10% of the certified values. The relative standard deviation for sediment samples in this study was less than 5%.

X-ray diffraction analyses were performed on selected samples using X-ray Powder Diffraction (XRD, MAC Science, MXP 3), which used a Cu target and was conducted at 45 kV and 40 mA, with a scanning step size 0.0083556°/s and a per time step of 1.27 s in the range of 2θ between 3° and 80°.

## Additional Information

**How to cite this article**: Ding, X. *et al*. Sulfur Geochemistry of a Lacustrine Record from Taiwan Reveals Enhanced Marine Aerosol Input during the Early Holocene. *Sci. Rep.* **6**, 38989; doi: 10.1038/srep38989 (2016).

**Publisher's note:** Springer Nature remains neutral with regard to jurisdictional claims in published maps and institutional affiliations.
